# Obesity Stigma: Causes, Consequences, and Potential Solutions

**DOI:** 10.1007/s13679-023-00495-3

**Published:** 2023-02-14

**Authors:** Susannah Westbury, Oyinlola Oyebode, Thijs van Rens, Thomas M. Barber

**Affiliations:** 1 School of Public Health and Preventative Medicine, Monash University, Melbourne, VIC Australia; 2 Wolfson Institute of Population Health, Queen Mary University of London, London, UK; 3 Department of Economics, University of Warwick, Coventry, UK; 4 Warwick Medical School, University of Warwick, Coventry, UK

**Keywords:** Public health, Obesity bias, Obesity stigma, Provider bias, Weight discrimination, Weight stigma

## Abstract

***Purpose of Review*:**

This review aims to examine (i) the aetiology of obesity; (ii) how and why a perception of personal responsibility for obesity so dominantly frames this condition and how this mindset leads to stigma; (iii) the consequences of obesity stigma for people living with obesity, and for the public support for interventions to prevent and manage this condition; and (iv) potential strategies to diminish our focus on personal responsibility for the development of obesity, to enable a reduction of obesity stigma, and to move towards effective interventions to prevent and manage obesity within the population.

***Recent Findings*:**

We summarise literature which shows that obesity stems from a complex interplay of genetic and environment factors most of which are outside an individual’s control. Despite this, evidence of obesity stigmatisation remains abundant throughout areas of media, entertainment, social media and the internet, advertising, news outlets, and the political and public health landscape. This has damaging consequences including psychological, physical, and socioeconomic harm.

***Summary*:**

Obesity stigma does not prevent obesity. A combined, concerted, and sustained effort from multiple stakeholders and key decision-makers within society is required to dispel myths around personal responsibility for body weight, and to foster more empathy for people living in larger bodies. This also sets the scene for more effective policies and interventions, targeting the social and environmental drivers of health, to ultimately improve population health.

## Introduction

Obesity is defined by The World Health Organisation as “*abnormal or excessive fat accumulation that may impair health*” [1]. The prevalence of obesity has risen exponentially over the last 50 years and is now so widespread that many have announced a state of obesity pandemic [2, 3]. In 2016, WHO estimated that globally, over 1.9 billion adults were overweight, including more than 650 million adults with obesity [1].

Obesity is a chronic relapsing disease characterised by an inflammatory state and associated with significant mortality and morbidity [4]. There are > 50 obesity-related conditions that include metabolic dysfunction (type 2 diabetes, hypertension, non-alcoholic fatty liver disease, polycystic ovary syndrome, and cardiovascular disease), mood disorders (depression and anxiety), dementia, joint problems (osteoarthritis), chronic kidney disease, obstructive sleep apnoea, and at least thirteen types of cancer [5–8]. Furthermore, obesity confers a substantial burden on psychological and psychosocial functioning, and has profound consequences on global health economic expenditure [9].

Obesity stigma is characterised by prejudiced, stereotyped, and discriminatory views and actions towards people with obesity, often fuelled by inaccurate ideas about the causes of obesity [9]. Despite decades of research supporting the dominant influence of genetic and environmental factors in the development of obesity, in the public consciousness, obesity continues to be viewed as a result of individual-level decision-making. This misperception leads to harmful assumptions about the lifestyles and characters of people with obesity. Such ensuing obesity stigma permeates our current sociocultural and political landscape and has severe consequences for people living with obesity, including worsened mental health [10•], increased mortality and morbidity [11, 12], and poor healthcare provision [13]. Furthermore, a narrative of personal responsibility for obesity development orientates individual-level interventions that are naïve to the reality of underlying genetic and environmental causes of obesity, that in turn receive inadequate attention and support.

Herein, we describe our current understanding of the aetiology of obesity and provide an overview of the evidence base for the impact of genetic and environmental factors. We examine the pervading focus on personal responsibility for obesity development and how this mindset leads to stigma; we explore the widespread and far-reaching consequences of obesity stigma. Finally, we conclude by reviewing promising potential strategies that would reframe obesity within the public consciousness and facilitate more effective and evidence-based interventions to improve both the prevention and management of obesity.

## Aetiology of Obesity

Our best current explanation for the global rise in the prevalence of obesity over recent decades promotes complex interactions between underlying genetic predisposition and our environment [14]. In essence, obesity results from a sustained positive energy balance in which excess calories are consumed, exceeding those that are expended [8]. It should be noted that this traditional view of the pathophysiology of obesity development is almost certainly an over-simplification, with important roles for hypothalamic regulation of appetite and energy expenditure, the effects of sugar consumption on such regulation (including both insulin and leptin resistance), and the complex interplay between such appetite and metabolism regulating pathways and the gut (including gut peptides, the autonomic nervous system, and the gut microbiota) [8]. These complex mechanisms, and the myriad ways in which our genes interact with environmental factors to influence body weight, remain incompletely understood [8].

Our brains and bodies are programmed to tightly regulate energy balance through both metabolic and hormonal systems that control appetite and satiety [8, 15]. For our ancestors, fat storage was necessary for survival, likely favouring gene variants that led to weight gain rather than weight loss [8, 16]. Key insights into such appetite-regulating genes stem from studies on single gene defects that strongly associate with obesity, including those in key genes such as proopiomelanocortin (POMC) and melanocortin 4 receptor (MC4R) [17] phenotypically characterised by intractable hunger and the development of severe obesity from an early age. However, such monogenic defects only affect a tiny proportion of the population with obesity, and cannot explain the recent global rise of obesity [16, 17].

Given that obesity is a heritable condition and monogenic gene defects only affect a small minority of people, it is important to consider the origin of the heritability of obesity [18]. Genome-wide association studies (GWAS) reveal common polygenic gene variants, for example in the fat mass and obesity-associated (FTO) gene region, that are associated with changes in fat mass and contribute towards the development of obesity [19]. However, even considering polygenic effects, these account for only ~ 3% of the heritability of obesity [20], whilst twin studies show that the real heritability potential of obesity is somewhere between 40 and 70% [21]. One explanation for the missing heritability stems from epigenetic and epigenomic factors, in which the expression of genes through transcription and translation is influenced through DNA methylation and histone modification. Such modifications to the DNA molecule are influenced heavily by gene–environment interactions, wherein our dietary, physical, in utero, and other environmental exposures activate or silence specific genes, influencing the central control of appetite and metabolism, and ultimately body weight [17, 22]. In essence, genetic predisposition to obesity manifests through gene–environmental interactions that underlie the pathophysiology of obesity.

In our evolutionary environment, caloric-restriction combined with a need for large amounts of physical activity (for example in the pursuit of prey, and to gather plant-based foods) a genetic predisposition for preserved body fat through appetitive and metabolic mechanisms would have been an advantage, benefitting those individuals and improving their survival (and reproductive) prospects during times of famine and other environmental threats [23]. In our modern-day obesogenic environments, such genetic predisposition for the preservation and deposition of fat within adipose tissue that so helped our evolutionary ancestors poses a great threat for modern-day hominids. In short, we are genetically maladapted to our modern-day environment [23].

Human biology helps to explain why we are seemingly so susceptible to environmental changes. Physiological processes regulating energy balance limit the extent to which individuals can “override” internal homeostatic systems and drivers to control their own body weight [24]. GWAS show that gene variants associated with BMI and food intake are mostly expressed in the central nervous system, particularly within the hypothalamus, and are therefore beyond conscious control [14]*.* In our obesogenic environment that promotes the desirability and availability of energy-dense food, combined with our modern-day society and culture that places so much emphasis on food and eating, it is exceedingly difficult for many individuals to defy the many automatic (including social, hedonic, and habitual) reflexes to eat, particularly when these are subconscious [24]. As put by Cohen et al., “*people have limited ability to shape the food environment individually and no ability to control automatic responses to food-related cues that are unconsciously perceived…*” [24]. This may help to explain the evidence for the difficulty experienced by many of losing and sustaining weight loss over a prolonged period [25, 26]. Although there are behaviour changes that individuals can implement to mitigate against weight gain and the development of obesity, at a population level, human weight seems largely at the mercy of our genetic makeup and environment.

Having considered genetic factors in the pathogenesis of obesity, it is important to consider the environmental contributors. The radical changes to our human environment over recent decades have rendered our neighbourhoods and daily lives almost unrecognisable even compared to 50 years ago. The environmental changes that most impact our propensity for weight gain and the development of obesity include those that influence our intake and expenditure of energy.

### Physical Activity

The technological revolution over the past 100 years has seen great changes to our physical world, characterised by mechanisation, computerisation, and automation [27]. Accordingly, there has been an unprecedented reduction in the need for humans to expend energy during the execution of everyday tasks that traditionally required physical exertion, for example, transportation and household chores [8, 28]. Trends in the built environment increasingly limit opportunities for physical activity through changes in urban landscape and design, poor neighbourhood walkability, and limited options for public transport [29–31]. Although data from the USA shows the percentage of people engaging in formal exercise (such as running, cycling, and strength training) has remained relatively stable over recent decades [28, 32], this accounts for only a small proportion of total daily energy expenditure, which is largely determined by occupation [33]. Workplace-related activity has steadily declined [32] alongside the rise of computer-based work-related tasks that involve sitting at a desk, increasing the proportion of the day spent sedentary [34]. This sedentary time is associated with overweight and obesity as well as insulin resistance, cardiovascular disease, and early mortality. Adverse relationships remain even for those who meet public health recommendations for moderate-to-vigorous physical activity [35, 36]. Other sedentary behaviours such as watching television, video games, and screen-time have increased in popularity, and also appear to promote the overconsumption of food [37]. In culmination, these trends lead to an overall reduction in energy expenditure.

### Global Food System

The global food system has shifted towards food that is increasingly processed, energy-dense, and nutrient-poor [27]. The “Western diet” is characterised by high levels of sugar and fat, high energy density, and low levels of fibre [8]. The marketing industry capitalises on human psychology in ways that maximise the efficacy of food promotion [27]. Further, increased commercial efficiency through mass production of energy-dense and highly processed foods has enabled affordability, whilst fresh and whole food produce such as fruits and vegetables have increased in price [38] which discourages a healthy diet [39]. In culmination, these trends have driven a large increase in energy consumption globally [40]. Between 1976 and 2000, The United States (US) Centers for Disease Control and Prevention (CDC) measured an increase in daily mean average energy intake of 179 kilocalories (7.3%) for men and 355 kilocalories (23.3%) for women [41]. This rise in energy intake mostly resulted from increased consumption of carbohydrates and sugary beverages [41, 42]. Other contributors to increasing caloric intake included changes to eating patterns, including increased snacking [43] (resulting primarily from the increased carbohydrate content of highly processed foods and the “rollercoaster” effects on blood sugar levels) and larger meal sizes [44]. These changes to eating behaviour increase the demand for food and therefore maximise the profits of the food industry [45]. These important changes in diet and eating behaviours coincided with a dramatic increase in the prevalence of obesity within the US population, which more than doubled from 14.5 to 30.9% in the same timespan [41].

Similar trends have been observed at different timepoints worldwide. In high-income countries, the transition to a positive energy balance began during the 1970s and 1980s [46]. A majority of middle-income countries and many low-income countries followed suit, particularly in the context of upward economically mobile populations [27]. Rapid urbanisation accelerated the rate of obesity prevalence in transitioning low- and middle-income countries, as evidenced by population-based data from Jamaica, Nigeria, and nations of the Pacific Islands. These geographical factors and time-trends affirm the strong impact of local physical and food environments on key behavioural drivers of obesity [8].

An important epidemiological consideration is that obesity does not affect populations equally, but rather disproportionately impacts underprivileged groups, most exposed to the environmental determinants of obesity, including rural populations, the poor, and minority ethnic groups. Recent findings from the Non-Communicable Disease (NCD) Risk Factor Collaboration [47] showed that overweight and obesity are greater in rural than urban areas in all high-income countries (HICs). Furthermore, in low- and middle-income countries (LMICs), the rate of increase of overweight and obesity is greater in rural than in urban settings. Indeed, the expected prevalence of overweight and obesity in rural settings may soon overtake that in urban areas [47, 48]. If this disproportionate burden of overweight and obesity in rural populations materialises globally, it will be compounded by additional challenges facing rural areas, including poverty, unemployment, worse healthcare access, lack of access to healthy, nutritious fresh produce, and insufficient public transport and infrastructure to facilitate physical activity [49].

Poverty has a complex relationship with overweight and obesity that varies according to country, income level, and type of income [50]. In HICs, obesity rates are highest amongst the poor [51]. In a large-scale study across the European older adult population, Salmasi and Celidoni showed low household income increased the probability of obesity by 0.146 for both men and women when controlled for key variables [52]. In contrast, overweight and obesity predominantly affects wealthier demographics in LMIC settings [53]. However, historical evidence suggests that as countries develop economically, the burden of obesity shifts towards the poorest people [51]. Unabated, these trends predict that in LMICs over the coming decades, the poorest population groups will experience the greatest rise in the prevalence of overweight and obesity [51].

In addition to socioeconomic status and wealth vs poverty, ethnic and racial groupings represent another important population-based contributor towards the development of obesity. In the USA between 2001 and 2002, African Americans, Native Americans, and Pacific Islanders had an obesity prevalence greater than 30%, whereas Asian Americans had an obesity prevalence of only 4.8% [54]. These disparities have multivariable and complex causes which are thought to include genetic variation [55, 56] as well as ethnic-specific rural location and poverty in addition to differences in healthcare access, social marginalisation, and behaviour [57]. The weight trajectories of new migrants provide insight into the impact of socioeconomic, sociocultural, and gene–environment interactions. Many migrants to HICs from LMICs arrive with a health advantage which includes healthier body weight than the native population; however, after 10–15 years post-migration, weight gain results in rates of overweight and obesity that often overtake the native population rate [58]. The influence of ethnic predisposition was highlighted in the Oslo Immigrant Health Study, wherein the prevalence of obesity amongst immigrants varied from 51% (Turkish) to 2.7% (Vietnamese) amongst the population [59].

#### Personal Responsibility as a Dominant Explanation for Obesity in Public Discourse

Disease stigma is a social phenomenon that occurs when distinct groups, often those with pre-existing vulnerabilities, are discriminated against on the basis of a medical condition, resulting in stereotyping, labelling, isolation, and reduced status. Ultimately, this results in discrimination [60]. There is a long and well-documented history of disease stigmatisation in public health history, towards conditions such as cholera, leprosy, tuberculosis, syphilis, drug addiction, mental illness, and perhaps most profoundly in recent memory, HIV/AIDs [9, 61].

Obesity stigma is characterised by negative and derogatory ideas about people with obesity. These stereotypes are closely linked to the concept that individuals with obesity are personally responsible for their own weight, despite a wealth of evidence as outlined above, that obesity largely reflects underlying genetic and environmental factors. Accordingly, assumptions are made about the character and behaviours of people with obesity, of being lazy, unhealthy, weak willed, greedy, glutinous and incompetent, and more broadly unclean, immoral, or otherwise defective [9, 62]. The result is societal endorsement of stigmatisation and discrimination of obesity that sees people with obesity amongst the last acceptable targets of prejudice, contempt, and ridicule. Current evidence suggests that obesity discrimination has increased exponentially over past decades [63], to a level that compared with racial discrimination in the USA by the first decade of this century [64].

**Fig. 1:**
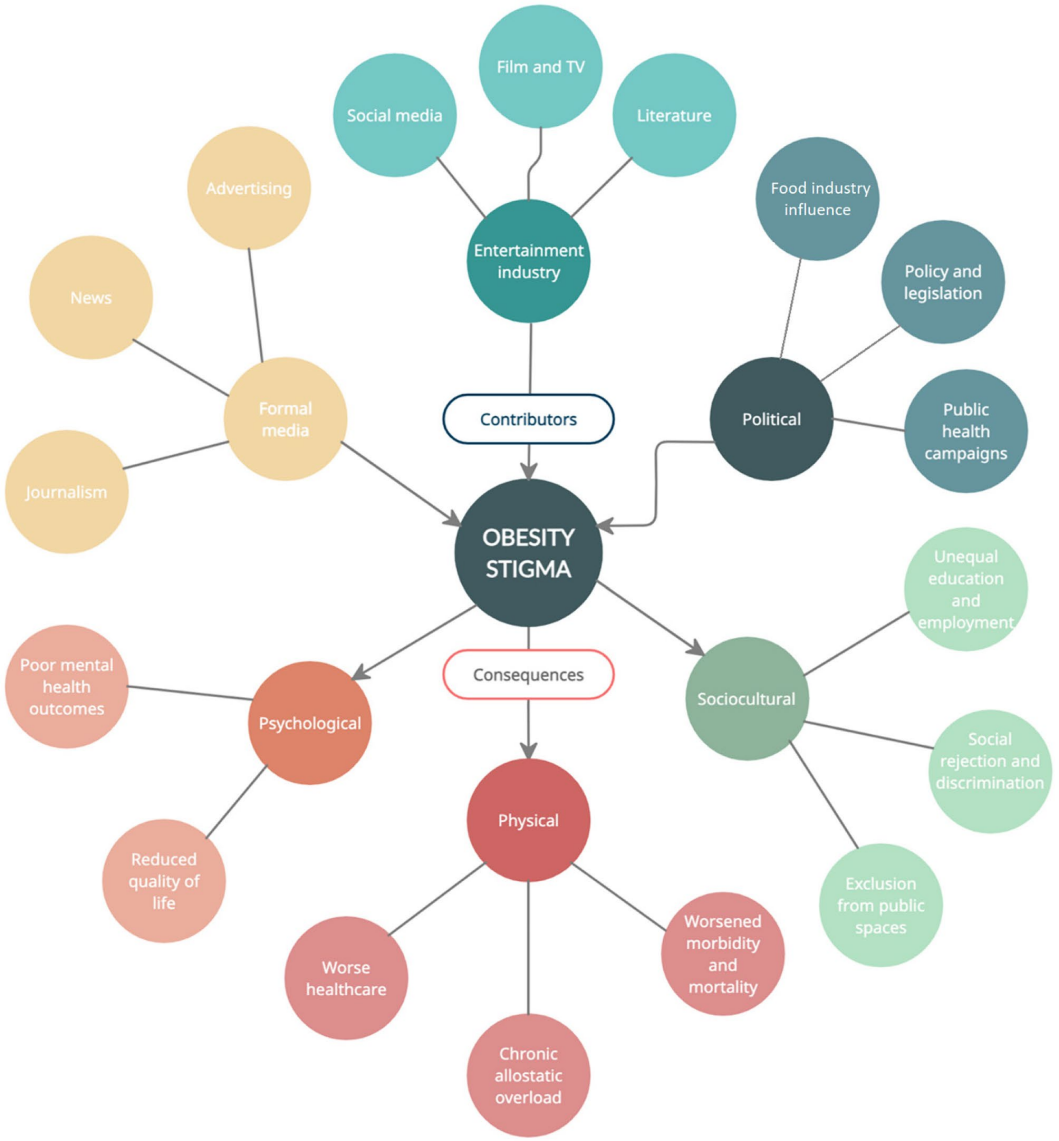
Contributors and consequences of obesity stigma

Evidence of obesity stigmatisation remains abundant throughout areas of media, entertainment, social media and the internet, advertising, news outlets, and the political and public health landscape. These drivers of obesity stigma are represented in Fig. 1. Within these domains, we review evidence of messages that affirm both obesity stigma and intertwined narrative of personal responsibility for obesity.

### Entertainment

The entertainment industry reinforces negative stereotypes of people with obesity through negative portrayals and underrepresentation. Characters with overweight and obesity are often portrayed as lonely, clownish, or misfits. Greenberg et al. found that of 1018 popular television show characters, overweight women were less likely to have romantic partners, display physical affection, or be considered attractive. Overweight men were less likely to have romantic partners or even friends and were more often shown eating. Furthermore, the representation of characters with overweight and obesity was less than half of that in the actual population, with only 25% of men and 14% of women having overweight or obesity [65]. Although Greenberg et al.’s work is now decades old, some of their findings have been repeated more recently [66]. This underrepresentation is consistent with a large quantity of research [62, 67–70]. In books and films, obesity is further stigmatised through the association of obesity with the portrayal of “evil” characters, a notable example being the Dursley family in JK Rowling’s *Harry Potter* series, who were abusive towards Harry throughout his childhood, and frequently filmed from angles that emphasised their weight and during snacking, tea, and biscuit or meal times. Concerningly, weight stigmatisation in television content seems to be particularly prominent in shows targeting adolescents, particularly girls [71]. Children’s media features frequent negative messages associated with people who have overweight and obesity, who are more often depicted as unattractive, friendless, unkind, and the “bad guy” compared with their normal-weight counterparts [67–69].

### Social Media

Social media is a leading source of news media and entertainment in the twenty-first century [72, 73]. Chou et al.’s mixed methods analysis of popular social media platforms including Twitter, Facebook, internet blogs, and forums revealed extensive evidence of negative stigmatisation of people with obesity characterised by derogation, exclusion, personal attacks, sexism, and misogyny. Alarmingly, cyberbullying, hostility, and verbal aggression particularly towards women with overweight and obesity were pervasive [74]. On YouTube, Yoo and Kim found that videos expressing derogatory views towards people with overweight and obesity tend to attract a high number of views, ratings, and viewer interaction. Personal accountability for obesity was a dominant rhetoric on YouTube, correlating with a preponderance of recommendations for changes in personal behaviour [75].

### Advertising

Obesity stigma is also perpetuated within the advertising and marketing industry, including the weight loss industry itself. Obesity has been exploited for economic gain in an industry valued at US $78 billion in the USA alone [76]. Paid advertisements both on commercial television and online traditionally portray people with overweight and obesity as both unattractive and unhappy, and focus exclusively on personal responsibility for obesity through promoting diet and exercise products [63]. Advertisers generally cultivate a belief that body weight is controllable through individual efforts and that leanness associates with success in all areas of life [77].

### News and Journalism

The depiction of obesity in the news and journalistic media outlets reinforces the stigmatisation of people with obesity [78]. McClure et al. showed that images negatively portraying people with obesity, for example depicting unflattering poses or stereotyped actions like eating fast food, promote obesity stigma [78]. In a study of the British press, Baker et al. showed that there was a doubling in the amount of reporting within newspapers on obesity between 2008 and 2017 [79•]. During this period, there was an increasing emphasis on individual responsibility and reduced focus on the social and political contributors towards obesity [79•]. Chiang et al. demonstrated similar trends across the USA between 2006 and 2015, wherein each year had a greater proportion of articles that discussed individual attribution for obesity compared with either environmental attributions or a mixed model of both individual and environmental attributions [80•]. This misrepresentation of the underlying contributors to the development of obesity reduces the societal perceived responsibility of governments and large corporations to address obesity [78].

### Government, Policy, and Legislation

Political action over the last two decades has galvanised policy and legislation *against* collective responsibility for the obesity epidemic, consistently enforcing personal responsibility for body weight. The food industry has pushed this framing of personal responsibility in policy debates [81–84]. In 2005, the US Congress proposed a “*Personal Responsibility in Food Consumption Act*” that served to protect the fast food industry from civil lawsuits resulting from weight gain [85]. Similar bills and legislations have subsequently been introduced in over 20 US states [86].

The rhetoric of personal responsibility for obesity is often touted by government officials. Former UK Prime Minister Tony Blair reported that the problem of obesity was “*not, strictly speaking, public health questions at all. They are questions of individual lifestyle…They are the result of millions of individual decisions*” [87]. Australian member of Parliament Ewen Jones claimed “*It’s not the government’s fault that I’m fat, it’s my fault and I live with the consequences*” [88].

It is no surprise then that public health campaigns, which are largely funded and guided by political support, often misrepresent obesity as a personal choice. Even when environmental and societal contributors are represented in discussion, solutions to obesity focus on changing *individual* behaviour in lieu of strategies consistent with the evidence base [78]. Demonstrative campaign titles include “*Pouring on the Pounds: Don’t Drink Yourself Fat*” [89] and the UK’s “*Choosing a Better Diet*” and “*Choosing Activity*” campaigns [90]. These public health efforts to reduce obesity also have stigmatising effects. Despite “positive” intentions, well-publicised campaigns have been called out for reinforcing prevailing negative attitudes towards and stereotypes of people with obesity [91–94]. Specific concerns include an intense focus on body shape and size in the context of an “ideal” body type [94].

### Body Positivity

It is worth mentioning the recent emergence of safe spaces for obesity-related issues and experiences that are free from judgement [95]. The body positivity and neutrality movements are two phenomena that reject narrow body ideals and focus on self-acceptance and respect for all body sizes [96, 97]. These movements seem to be making progress in the representation of people with obesity in the media. Advertising campaigns that promote body acceptance appear to increase self-esteem and mood [98], and the use of average and plus-sized models tend to reduce body-focussed anxiety and improve body satisfaction of viewers [99]. Groups within social media and the internet are creating digital spaces where obesity stigma is challenged and people with obesity are included and empowered, having a voice that is rarely represented in the physical world [95, 100].

These positive changes may suggest that we are amid a transitional period for the representation of people with obesity within our society. However, progress does not appear to be occurring across all domains, particularly in news, political, and public health media. Furthermore, the examples outlined here reflect a likely minority of trends in the representation of obesity, and obesity stigmatisation continues to appear rife within the public consciousness and lived experience of people with obesity.

## Consequences of Obesity Stigma

The damaging effects of obesity stigmatisation are widespread and include psychological, physical, and socioeconomic harm [Fig. 1]. Strong evidence supports obesity stigma as an important contributor to poor mental health outcomes for people living with obesity, who are 32% more likely to develop depression compared with their normal-weight counterparts [101]. A recent large meta-analysis synthesising 105 studies including data on > 59,000 participants found perceived obesity stigma amongst individuals was associated significantly with poorer mental health (*r* =  − 0.35, *p* ≤ 0.001), which remained significant following adjustment for relevant variables including body weight [10•]. These data suggest that depression associates with obesity stigmatisation rather than obesity per se. Perceived obesity stigma also had a strong effect on body image dissatisfaction, quality of life, dysfunctional eating, and severity of depression or anxiety symptoms [10•]. There is also evidence that internalised stigma, often referred to as obesity “self-stigma” or “weight bias internalization” (WBI), associates with similar negative mental health outcomes compared with externally based obesity stigma [102]. A recent meta-analysis by Alimoradi et al. revealed a similar moderate-large effect size for weight-related self-stigma and psychological distress (corrected Fisher’s *Z*: depression = 0.40; anxiety = 0.36) [103].

Beyond its severe mental health consequences, obesity stigma is also detrimental to short- and long-term physical health. Counter to traditional public health beliefs that social pressure encourages people with obesity to lose weight [25], ironically, evidence suggests that obesity stigma actually increases the risk of obesity. Obesity stigma may be associated with increased difficulty of losing weight and medication non-adherence and people with obesity may exclude themselves from some exercise settings [102, 104]. Pearl and Puhl’s systematic review found that obesity self-stigma is associated with worse dietary adherence and reduced motivation and self-efficacy to complete health-promoting behaviours [105•]. Unlike other public health issues addressing social norms, such as tobacco smoking [106], making obesity socially unacceptable does not appear to reduce obesity rates, and on the contrary results in increased harms.

In addition to worsening mental and physical health, obesity stigma may also augment all-cause mortality and shorten lifespan. Amongst participants from two large longitudinal studies in the USA, those who experienced weight stigma and discrimination had an increased mortality of almost 60% (The Health and Retirement Study, hazard ratio = 1.57, 95% CI: 1.34–1.84; Midlife in the United States Study, hazard ratio = 1.59, 95% CI = 1.09–2.31). This increased mortality risk persisted when controlled for common risk factors, including BMI [11]. Chronic psychological stress resulting from obesity stigma can trigger activation of the hypothalamo-pituitary adrenal axis with increased release of adrenally derived cortisol that in turn can drive increased fat deposition and appetite [107, 108]. Enhanced cortisol release may contribute to increased mortality through weight gain and associations with inflammation, immune dysregulation, hypertension, insulin resistance, and oxidative stress [109–111]. Furthermore, enhanced cortisol release may also mediate some of the worsening effects of obesity stigma on abdominal obesity, glycaemic control, and the development of metabolic syndrome [12]. These associations parallel the pathophysiology contributing to worse health outcomes for those experiencing other forms of discrimination such as racism [112, 113].

Obesity stigma contributes to poorer healthcare for people with obesity. There is growing evidence that healthcare providers have strong explicit and implicit biases against people with obesity [109, 114]. Healthcare obesity stigma is characterised by stereotypes of laziness, lack of discipline, and willpower [115]. Inevitably, this mindset influences the judgement, behaviour, and decision-making of healthcare providers [115], who tend to have less respect for people with obesity [116] and believe that people with obesity are less likely to follow self-care recommendations or adhere to recommended treatments [117, 118]. Healthcare providers have also been more likely to perceive the care of people with obesity as a “waste of time” [115], and are known to spend less time in consultations with people with obesity than their normal-weight counterparts [119, 120]. Other healthcare issues that have previously reported stem from obesity stigma include the over-attribution of symptoms to obesity, failure to explore alternate diagnoses, reduced exploration of treatment options (“therapeutic inertia”), and hesitancy to conduct clinical examinations [121, 122].

Understandably, people with obesity have reported avoiding healthcare encounters due to discriminatory and stigmatising experiences [123, 124]. People with obesity report being mistreated and even ignored when receiving healthcare, and are up to three times more likely to report being denied healthcare [13]. Obesity stigma within healthcare and stigmatised judgements from healthcare professionals also perpetuates obesity by reducing the likelihood of people achieving their weight loss targets [125].

Finally, the socioeconomic impact of obesity is extensive. In employment, researchers from high-income countries believe that having obesity negatively impacts wages, promotion, and the potential for disciplinary action [126, 127]. In the USA, people with obesity have previously been found to be less likely to be hired than their lean counterparts, even when qualifications are identical [128]. In Korea, women who are overweight receive less pay than lean women for the same work [129]. There is plentiful anecdotal evidence of people getting fired for having overweight or obesity [130, 131]. In education also, obesity stigma appears to be present at all levels of schooling and college — at least in some countries — and leads to prejudice, rejection, and harassment, making educational spaces less safe for people with obesity [126]. In public settings too such as theatres, cinemas, shops, restaurants, and transport, obesity stigma may shape attitudes that people with obesity should not be accommodated for. Accordingly, people with obesity may be prevented from the same level of participation as their lean counterparts through a public infrastructure that fails to accommodate them adequately. Overall, obesity stigma has a substantial impact on socioeconomic factors through diverse means that include unequal standards in education, employment, career progression, salary, and public infrastructure.

## Solutions

### Why?

Addressing obesity stigma is a healthcare imperative. As outlined, obesity stigma has severe consequences for people living with obesity, including but not limited to psychological distress, mental illness, increased mortality and morbidity, and worse healthcare [10•, 11–13]. Furthermore, obesity stigma perpetuates obesity through physiological, psychological, and social effects, acting like a vicious circle [25].

Addressing obesity stigma is also an ethical imperative. Stigma burdens groups with undue discrimination, prejudice, and exclusion, and dehumanises them in the face of their community [60]. Burris argued that stigma evoked “*the total destruction of the individual’s status in organized society. It is a form of punishment more primitive than torture*” [132]. Stigma is especially unethical in the context of obesity insofar that it burdens already underprivileged and vulnerable groups, such as the global poor, rural, and certain minority ethnic groups [60].

Addressing obesity stigma is necessary to improve the public health efforts to prevent and manage obesity, which despite global efforts has had limited success to date [133]. Interventions that target the individual have had little success, partly due to obesity stigma-induced barriers to the widespread adoption of healthy behaviours [133, 134]. When obesity is seen as a personal choice, as reinforced by obesity stigma, solutions focus on changing individual behaviours in lieu of synergistic strategies that focus on changing systems and environments to support healthy behaviours, the latter being consistent with the current evidence base [25, 78, 133, 135, 136]. However, such an approach is hampered through widespread obesity stigma within society. Re-calibrating this perception amongst society, including politicians, healthcare providers, and town planners, will help to support the development of effective public health strategies for the future that should properly address the many and diverse environmental and systemic contributors to the development of obesity, balanced with consideration of personal factors.

### How?

Firstly, it is important to acknowledge the striking paucity of research on the topic of reducing obesity stigma within society. Authors of systematic reviews have repeatedly highlighted this deficiency and the low quality of existing research papers within the field [137–139]. This scenario may reflect the early stages of this field and/or a lack of interest, perhaps stemming from an acceptance of obesity stigma (including amongst researchers). A prerequisite for tackling the problem of obesity stigma within society is the generation of high-quality research on effective interventions that have consistent theoretical frameworks, strong study designs, and sound methodologies [137, 138]. Such data will facilitate the development of a consensus on the development of optimal strategies to reduce obesity stigma within society, and enable implementation of consistent and co-ordinated public health action [138].

Secondly, shifting public health messaging away from obesity and towards healthy behaviours, or alternatively away from behaviour completely, to allow the appropriate focus on the environments where the behaviour takes place, may facilitate the deconstruction of obesity stigma. We do not deny that there is strong evidence that having overweight and obesity increases all-cause mortality [140], and that weight loss can improve obesity-related morbidity [141]. However, benefits of healthy behaviours are often overlooked in the context of BMI [142]. The 15-year prospective Rotterdam study showed that physical activity moderated the risk of cardiovascular disease in people with overweight and obesity to the extent that there was no difference in CVD risk between people with high or normal-range BMI [143]. Other studies show that healthy diets may reduce all-cause mortality risk, particularly CVD risk, even when accounting for BMI [144].

Based on such evidence, placing too much emphasis on obesity per se, and focussing too much on weight loss purely to reduce obesity severity, is perhaps unhelpful. Although this may appear counterintuitive, such a traditional approach to obesity unfortunately also places emphasis on appearance and may actually demotivate and ostracise people with obesity [9, 145], thereby hampering rather than helping with obesity management. An alternate approach, and one that we support, promotes the use of public health policies that encourage the adoption of healthy behaviours, including for example nutrient-rich diets cooked from their raw ingredients (rather than ultra-processed foods), regular engagement in physical activity, and sleep sufficiency by intervening to create environmental drivers for these behaviours. All people, including those with overweight and obesity, should be empowered and supported through structural interventions and policies and positive public health messaging to adopt such healthy lifestyle activities and behaviours [146, 147]. This approach does not deny the harmful effects of excess body weight, but by detracting attention from body shape and size should help to diminish societal obesity stigma, whilst facilitating healthy living, that in turn should help in the prevention and management of obesity, stigma-free.

As interventions that rely solely on education and individual behaviour change are largely ineffective [148, 149], enabling healthy behaviours will require both physical and food environmental changes and fiscal policies to support them [25]. Examples include improvements to the availability, accessibility, and affordability of fresh nutrient-rich foods, improved public transportation and urban planning to facilitate active and safe outdoor lifestyles [150, 151]. Importantly, improving the “healthfulness” of our food and physical environments should also help to establish improved equity in the distribution of key resources within the population.

Thirdly, deconstructing obesity stigma through educational interventions is promising. Educational interventions that provide information on the genetic and environmental causes of obesity have shown some success in changing attitudes about how much control individuals have over their own body weight [139, 152]. Other studies on healthcare students and workers have had modest success by evoking empathy and acceptance of persons with obesity through positive contact [139]. Current evidence suggests that the greatest efficacy on tackling obesity stigma is achieved when multiple and diverse educational strategies are combined [139].

Extrapolating these early findings, obesity could be reframed in public education efforts as a chronic condition that manifests primarily from a combination of genetic predisposition that interacts maladaptively with our obesogenic environment: factors that are predominantly beyond our individual control [9]. Furthermore, people living with obesity should receive positive representation in the media, including acceptance, inclusion, and empowerment. Importantly, the voices of people with obesity should be amongst the forefront of these public health campaigns [153]. This conceptual overhaul will require significant and sustained public education efforts that incorporate “top-down” and “bottom-up” approaches, such as education within schools and universities, and efforts to re-define obesity and its causes within government and industry [134]. Educational efforts could be targeted at institutions where the impact of obesity stigma is particularly pronounced, such as healthcare, educational settings, and places of employment [110].

The re-classification of obesity has been discussed by others as key to education efforts. Obesity was labelled a disease by the WHO International Classification of Diseases, the American Medical Association, and the World Obesity Federation in the early 2000–2010’s [154]. There is significant debate in academic and public realms on the appropriateness of this stance [154]. Some argue a disease label “legitimises” obesity by acknowledging biological and genetic underpinnings, and could increase attention and resource allocation to obesity research, prevention, and treatment [156]. In contrast, there is legitimate concern that a disease label will worsen the stigmatisation of people with obesity and increase discrimination [155, 157]. There is also evidence that disease-labelling may disempower and reduce self-efficacy; Hoyt et al. found that labelling obesity as a disease reduced concerns about weight and predicted higher-calorie food choices amongst people with higher BMIs [158]. We caution against the labelling of obesity as a disease prior to more extensive investigation of its impact on obesity stigmatisation and psychosocial wellbeing, in addition to potential policy, fiscal, and healthcare impacts.

Fourthly, efforts to reduce obesity stigmatisation in the public domain could be spearheaded by legislation to prohibit prejudice and discrimination on the basis of weight [86]. Although educational efforts are important, without the support of our formal institutions, these messages are likely to be insufficient [159]. Few national or state legislations globally protect citizens from weight discrimination, providing legal freedom for industries to discriminate based on obesity status [62]. Weight-based discrimination should be formally recognised as a legitimate social concern and be included in antidiscrimination acts that prohibit discrimination based on other personal characteristics such as sex, marital status, or disability. Notably, it will be important to balance the need for protection and equal treatment of people with obesity against the risk of even greater obesity stigmatisation that may stem from such new legislation [160]. Position statements from government and public health organisations should demonstrate non-stigmatising language and discourse around obesity. There is support for this approach from people enrolled in an international weight-management programme across six countries [161•].

Implementing these changes will take no less than a social overhaul and is likely to require decades of consistent action. However, the promise of change is already being seen through the body positivity movements and the creation of “safe spaces” for obesity in certain domains. Perhaps we can use the example of racial discrimination, which decades ago was rife globally, and in many countries acceptable and legally permitted and even encouraged through, for example, apartheid. Although, sadly, racial discrimination continues in our modern world, it is often illegal, and generally much better recognised and managed than in previous decades. We need to move towards such a scenario with obesity stigma and discrimination.

## Conclusion

We predict that in the decades to come, we will look back at our current era in shame. We will recognise obesity stigma for what it is: discrimination just like any other form of discrimination that has become normalised within our society to an extent that its existence often even goes unnoticed. An important step on this long road will be to dispel myths around obesity, and to educate society on its true causes. Improved understanding should help to dispel associated myths around personal responsibility and should help to foster more empathy for people living with obesity. Gradually, such renewed understanding and insights should help us to have the courage and conviction to question obesity stigma when we encounter it, and hold the perpetrators to account, so that they too can question their misjudged beliefs and behaviours. As outlined, this approach will only work through a combined, concerted, and sustained effort from multiple stakeholders and key decision-makers within society. Only then can we hope for a transformed society which is finally freed from the shackles of obesity stigma, in which body weight no longer defines the people living in it.

## References

[1] World Health Organization. Obesity and overweight. https://www.who.int/news-room/fact-sheets/detail/obesity-and-overweight. Accessed 2021.

[2] Meldrum DR, Morris MA, Gambone JC (2017). Obesity pandemic: causes, consequences, and solutions—but do we have the will?. Fertil Steril.

[3] The Lancet Gastroenterology and Hepatology (2021). Obesity: another ongoing pandemic. The Lancet Gastroenterology & Hepatology: Editorial.

[4] Bray GA, Kim KK, Wilding JPH. Obesity: a chronic relapsing progressive disease process. A position statement of the World Obesity Federation. Obes Rev 2017, 18(7):715–723. 10.1111/obr.12551 28489290

[5] Lauby-Secretan B, Scoccianti C, Loomis D, Grosse Y, Bianchini F, Straif K (2016). International Agency for Research on Cancer Handbook Working G: Body fatness and cancer—viewpoint of the IARC Working Group. N Engl J Med.

[6] Singh GM, Danaei G, Farzadfar F, Stevens GA, Woodward M, Wormser D, Kaptoge S, Whitlock G, Qiao Q, Lewington S (2013). The age-specific quantitative effects of metabolic risk factors on cardiovascular diseases and diabetes: a pooled analysis. PLoS ONE.

[7] Hruby A, Hu FB (2015). The epidemiology of obesity: a big picture. Pharmacoeconomics.

[8] Blüher M (2019). Obesity: global epidemiology and pathogenesis. Nat Rev Endocrinol.

[9] Puhl RM, Heuer CA (2010). Obesity stigma: important considerations for public health. Am J Public Health.

[10] • Emmer C, Bosnjak M, Mata J. The association between weight stigma and mental health: a meta-analysis. Obesity Reviews 2020, 21(1):e12935. **A meta-analysis of 105 studies published up to 2019 found a medium to large negative association between weight stigma and mental health. There was a lot of heterogeneity in effect size between studies, with further research needed to explain this.** 10.1111/obr.12935 31507062

[11] Sutin AR, Stephan Y, Terracciano A (2015). Weight discrimination and risk of mortality. Psychol Sci.

[12] Tsenkova VK, Carr D, Schoeller DA, Ryff CD (2011). Perceived weight discrimination amplifies the link between central adiposity and nondiabetic glycemic control (HbA1c). Ann Behav Med.

[13] Carr D, Friedman MA (2005). Is obesity stigmatizing? Body weight, perceived discrimination, and psychological well-being in the United States. J Health Soc Behav.

[14] Berthoud H-R, Münzberg H, Morrison CD (2017). Blaming the brain for obesity: integration of hedonic and homeostatic mechanisms. Gastroenterology.

[15] Locke AE, Kahali B, Berndt SI, Justice AE, Pers TH, Day FR, Powell C, Vedantam S, Buchkovich ML, Yang J (2015). Genetic studies of body mass index yield new insights for obesity biology. Nature.

[16] Baqai N, Wilding JPH (2015). Pathophysiology and aetiology of obesity. Medicine.

[17] Thaker VV (2017). Genetic and epigenetic causes of obesity. Adolesc Med State Art Rev.

[18] Waalen J (2014). The genetics of human obesity. Transl Res.

[19] Fawcett KA, Barroso I (2010). The genetics of obesity: FTO leads the way. Trends in genetics : TIG.

[20] Albuquerque D, Nóbrega C, Manco L, Padez C (2017). The contribution of genetics and environment to obesity. Br Med Bull.

[21] Barsh GS, Farooqi IS, O’Rahilly S (2000). Genetics of body-weight regulation. Nature.

[22] Fall T, Mendelson M, Speliotes EK (2017). Recent advances in human genetics and epigenetics of adiposity: pathway to precision medicine?. Gastroenterology.

[23] Lakka H-M, Bouchard C. Chapter 3 — etiology of obesity. In: Surgical management of obesity. edn. Edited by Buchwald H, Cowan GSM, Pories WJ. Philadelphia: W.B. Saunders; 2007: 18–28.

[24] Cohen DA (2008). Neurophysiological pathways to obesity: below awareness and beyond individual control. Diabetes.

[25] Vartanian LR, Smyth JM (2013). Primum non nocere: obesity stigma and public health. Journal of Bioethical Inquiry.

[26] Dansinger ML, Gleason JA, Griffith JL, Selker HP, Schaefer EJ (2005). Comparison of the Atkins, Ornish, Weight Watchers, and Zone diets for weight loss and heart disease risk reduction: a randomized trial. JAMA.

[27] Swinburn BA, Sacks G, Hall KD, McPherson K, Finegood DT, Moodie ML, Gortmaker SL (2011). The global obesity pandemic: shaped by global drivers and local environments. Lancet.

[28] Malik VS, Willett WC, Hu FB (2013). Global obesity: trends, risk factors and policy implications. Nat Rev Endocrinol.

[29] Andreyeva T, Blumenthal DM, Schwartz MB, Long MW, Brownell KD (2008). Availability and prices of foods across stores and neighborhoods: the case of New Haven. Connecticut Health Aff (Millwood).

[30] Booth KM, Pinkston MM, Poston WS (2005). Obesity and the built environment. J Am Diet Assoc.

[31] Sallis JF, Saelens BE, Frank LD, Conway TL, Slymen DJ, Cain KL, Chapman JE, Kerr J (2009). Neighborhood built environment and income: examining multiple health outcomes. Soc Sci Med.

[32] Brownson RC, Boehmer TK, Luke DA (2004). Declining rates of physical activity in the United States: what are the contributors?. Annu Rev Public Health.

[33] Church T, Martin CK (2018). The obesity epidemic: a consequence of reduced energy expenditure and the uncoupling of energy intake?. Obesity.

[34] Parry S, Straker L (2013). The contribution of office work to sedentary behaviour associated risk. BMC Public Health.

[35] Healy GN, Wijndaele K, Dunstan DW, Shaw JE, Salmon J, Zimmet PZ, Owen N (2008). Objectively measured sedentary time, physical activity, and metabolic risk. Diabetes Care.

[36] Healy GN, Dunstan DW, Salmon J, Cerin E, Shaw JE, Zimmet PZ, Owen N (2008). Breaks in sedentary time: beneficial associations with metabolic risk. Diabetes Care.

[37] Chaput JP, Klingenberg L, Astrup A, Sjödin AM (2011). Modern sedentary activities promote overconsumption of food in our current obesogenic environment. Obes Rev.

[38] Finkelstein EA, Ruhm CJ, Kosa KM (2005). Economic causes and consequences of obesity. Annu Rev Public Health.

[39] Pancrazi R, van Rens T, Vukotic M. How distorted food prices discourage a healthy diet. Science Advances. 2022;8(13). 10.1126/sciadv.abi8807 PMC11093214 35353561

[40] Vasileska A, Rechkoska G (2012). Global and regional food consumption patterns and trends. Procedia Soc Behav Sci.

[41] The Centers for Disease Control and Prevention (2004). Trends in intake of energy and macronutrients—United States, 1971–2000. MMWR Morb Mortal Wkly Rep.

[42] Putnam J, Allshouse J, Kantor LS (2002). US per capita food supply trends: more calories, refined carbohydrates, and fats. Food Review.

[43] Nielsen SJ, Popkin BM (2003). Patterns and trends in food portion sizes, 1977–1998. JAMA.

[44] Young LR, Nestle M (2002). The contribution of expanding portion sizes to the US obesity epidemic. Am J Public Health.

[45] Chan RS, Woo J (2010). Prevention of overweight and obesity: how effective is the current public health approach. Int J Environ Res Public Health.

[46] Sassi F, Devaux M, Cecchini M, Rusticelli E. The obesity epidemic: analysis of past and projected future trends in selected OECD countries. OECD Health Working Papers No. 45. In: OECD Health Working Papers No 45. France: OECD. 2009:82.

[47] Bixby H, Bentham J, Zhou B, Di Cesare M, Paciorek CJ, Bennett JE, Taddei C, Stevens GA, Rodriguez-Martinez A, Carrillo-Larco RM (2019). Rising rural body-mass index is the main driver of the global obesity epidemic in adults. Nature.

[48] Popkin B. Rural areas drive increases in global obesity. In*.* 2019. 10.1038/d41586-019-01182-x 31068717

[49] Cohen SA, Greaney ML, Sabik NJ (2018). Assessment of dietary patterns, physical activity and obesity from a national survey: rural-urban health disparities in older adults. PLoS ONE.

[50] Mathieu-Bolh N. The elusive link between income and obesity. Journal of Economic Surveys 2021, n/a(n/a).

[51] Templin T (2019). Cravo Oliveira Hashiguchi T, Thomson B, Dieleman J, Bendavid E: The overweight and obesity transition from the wealthy to the poor in low- and middle-income countries: a survey of household data from 103 countries. PLoS Med.

[52] Salmasi L, Celidoni M (2017). Investigating the poverty-obesity paradox in Europe. Econ Hum Biol.

[53] Bukhman G, Mocumbi AO, Atun R, Becker AE, Bhutta Z, Binagwaho A, Clinton C, Coates MM, Dain K, Ezzati M (2020). The Lancet NCDI Poverty Commission: bridging a gap in universal health coverage for the poorest billion. The Lancet.

[54] Wang Y, Beydoun MA (2007). The obesity epidemic in the United States—gender, age, socioeconomic, racial/ethnic, and geographic characteristics: a systematic review and meta-regression analysis. Epidemiol Rev.

[55] Sankar P, Cho MK, Condit CM, Hunt LM, Koenig B, Marshall P, Lee SS, Spicer P (2004). Genetic research and health disparities. JAMA.

[56] Fesinmeyer MD, North KE, Ritchie MD, Lim U, Franceschini N, Wilkens LR, Gross MD, Bůžková P, Glenn K, Quibrera PM (2013). Genetic risk factors for BMI and obesity in an ethnically diverse population: results from the population architecture using genomics and epidemiology (PAGE) study. Obesity.

[57] Stryjecki C, Alyass A, Meyre D (2018). Ethnic and population differences in the genetic predisposition to human obesity. Obes Rev.

[58] Murphy M, Robertson W, Oyebode O (2017). Obesity in international migrant populations. Curr Obes Rep.

[59] Kumar BN, Meyer HE, Wandel M, Dalen I, Holmboe-Ottesen G (2006). Ethnic differences in obesity among immigrants from developing countries, in Oslo. Norway Int J Obes (Lond).

[60] Link BG, Phelan JC (2001). Conceptualizing stigma. Ann Rev Sociol.

[61] Bayer R (2008). Stigma and the ethics of public health: not can we but should we. Soc Sci Med.

[62] Puhl RM, Heuer CA (2009). The stigma of obesity: a review and update. Obesity.

[63] Andreyeva T, Puhl RM, Brownell KD (2008). Changes in perceived weight discrimination among Americans, 1995–1996 through 2004–2006. Obesity (Silver Spring).

[64] Puhl RM, Andreyeva T, Brownell KD (2008). Perceptions of weight discrimination: prevalence and comparison to race and gender discrimination in America. Int J Obes (Lond).

[65] Greenberg BS, Eastin M, Hofschire L, Lachlan K, Brownell KD (2003). Portrayals of overweight and obese individuals on commercial television. Am J Public Health.

[66] Mastro D, Figueroa-Caballero A. Measureing extremes: a quantitative content analysis of prime time TV depictions of body type. J Broadcast Electron media. 2018;62(2).

[67] Robinson T, Callister M, Jankoski T (2008). Portrayal of body weight on children’s television sitcoms: a content analysis. Body Image.

[68] Klein H, Shiffman KS (2006). Messages about physical attractiveness in animated cartoons. Body Image.

[69] Klein H, Shiffman KS (2005). Thin is “in” and stout is “out” what animated cartoons tell viewers about body weight. Eat Weight Disord.

[70] Fouts G, Vaughan K (2002). Television situation comedies: male weight, negative references, and audience reactions. Sex Roles.

[71] Eisenberg ME, Carlson-McGuire A, Gollust SE, Neumark-Sztainer D (2015). A content analysis of weight stigmatization in popular television programming for adolescents. Int J Eat Disord.

[72] Auxier B, Anderson M. Social media use in 2021. Pew Research Center 2021.

[73] Shearer E, Mitchell A. News use across social media platforms in 2020. 2021.

[74] Chou W-YS, Prestin A, Kunath S. Obesity in social media: a mixed methods analysis. Transl Behav Med. 2014;4(3):314–323. 10.1007/s13142-014-0256-1 PMC4167901 25264470

[75] Yoo JH, Kim J (2012). Obesity in the new media: a content analysis of obesity videos on YouTube. Health Commun.

[76] Market Data Enterprises. The U.S. weight loss & diet control market [https://www.giiresearch.com/report/md996330-us-weight-loss-diet-control-market-16th-edition.html?] Accessed 2021.

[77] Mishra S (2017). From self-control to self-improvement: evolving messages and persuasion techniques in weight loss advertising (1930–1990). Vis Commun.

[78] McClure KJ, Puhl RM, Heuer CA (2011). Obesity in the news: do photographic images of obese persons influence antifat attitudes?. J Health Commun.

[79] • Baker P, Brookes G, Atanasova D, Flint SW. Changing frames of obesity in the UK press 2008–2017. Soc Sci Med. 2020;264:113403. **This study examined every article in the UK press published between 2008 and 2017 containing the word “obese” or “obesity” and used computational methods to identify the way obesity is framed in the articles. The amount of press attention for obesity has grown over time, as have trends that frame obesity as a biomedical problem as well as the responsibility of individuals and their lifestyles.**

[80] • Chiang J, Arons A, Pomeranz JL, Siddiqi A, Hamad R. Geographic and longitudinal trends in media framing of obesity in the United States. Obesity. 2020;28(7):1351–1357. **This study examined every article in the US press published between 1006 and 2015 that mentioned the term “obesity” and used computational methods to categorise articles as attributing obesity to individual level causes, environmental causes, both, or none. The study found that the proportion of articles focused on individual level causes of obesity grew over time.** 10.1002/oby.22845 PMC7311269 32475076

[81] Williams S, Hill SE, Oyebode O (2022). *‘Choice should be made through… educated decisions not regressive dictates’*: discursive framings of a proposed ‘sugar tax’ in Bermuda: analysis of submissions to a government consultation. Global Health.

[82] Mialon M, Swinburn B, Allender S (2016). Systematic examination of publicly-available information reveals the diverse and extensive corporate political activity of the food industry in Australia. BMC Public Health.

[83] Anaf J, Fisher M, Handsley E, Baum F, Friel S (2021). ‘Sweet talk’: framing the merits of a sugar tax in Australia. Health Promot Int.

[84] Ngqangashe Y, Cullerton K, Phulkerd S, Huckel Schneider C, Thow AM, Friel S. Discursive framing in policies for restricting the marketing of food and non-alcoholic beverages Food Policy. 2022:102270. 10.1016/j.foodpol.2022.102270.

[85] Burnett D (2006). Fast-food lawsuits and the cheeseburger bill: critiquing Congress’s response to the obesity epidemic. Va J Soc Pol’y & L.

[86] Pomeranz JL (2008). A historical analysis of public health, the law, and stigmatized social groups: the need for both obesity and weight bias legislation. Obesity.

[87] Martin R (2008). The role of law in the control of obesity in England: looking at the contribution of law to a healthy food culture. Australia and New Zealand health policy.

[88] Marszalek J. MP Ewen Jones has demanded a healthy food rating system be dropped. In: Herald Sun. Melbourne 2014.

[89] NYC Department of Health and Mental Hygiene. Health bulletin: pouring on the pounds. In*.*, vol. 6:8. New York: NYC Health; 2009.

[90] Theis DRZ, White M (2021). Is obesity policy in England fit for purpose? Analysis of government strategies and policies, 1992–2020. Milbank Q.

[91] Puhl R, Luedicke J, Lee Peterson J (2013). Public reactions to obesity-related health campaigns: a randomized controlled trial. Am J Prev Med.

[92] Stein K (2008). Obesity PSAs: are they working as a public service?. J Am Diet Assoc.

[93] Katz DL, Murimi M, Pretlow RA, Sears W (2012). Exploring effectiveness of messaging in childhood obesity campaigns. Child Obes.

[94] Walls HL, Peeters A, Proietto J, McNeil JJ (2011). Public health campaigns and obesity — a critique. BMC Public Health.

[95] Dickins M, Thomas SL, King B, Lewis S, Holland K (2011). The role of the fatosphere in fat adults’ responses to obesity stigma: a model of empowerment without a focus on weight loss. Qual Health Res.

[96] Lazuka RF, Wick MR, Keel PK, Harriger JA (2020). Are we there yet? Progress in depicting diverse images of beauty in Instagram’s body positivity movement. Body Image.

[97] Cohen R, Irwin L, Newton-John T, Slater A (2019). #bodypositivity: a content analysis of body positive accounts on Instagram. Body Image.

[98] Selensky JC, Carels RA (2021). Weight stigma and media: an examination of the effect of advertising campaigns on weight bias, internalized weight bias, self-esteem, body image, and affect. Body Image.

[99] Clayton RB, Ridgway JL, Hendrickse J (2017). Is plus size equal? The positive impact of average and plus-sized media fashion models on women’s cognitive resource allocation, social comparisons, and body satisfaction. Commun Monogr.

[100] Zavattaro SM (2021). Taking the social justice fight to the cloud: social media and body positivity. Public Integrity.

[101] Pereira-Miranda E, Costa PRF, Queiroz VAO, Pereira-Santos M, Santana MLP (2017). Overweight and obesity associated with higher depression prevalence in adults: a systematic review and meta-analysis. J Am Coll Nutr.

[102] Papadopoulos S, Brennan L (2015). Correlates of weight stigma in adults with overweight and obesity: a systematic literature review. Obesity.

[103] Alimoradi Z, Golboni F, Griffiths MD, Broström A, Lin C-Y, Pakpour AH (2020). Weight-related stigma and psychological distress: a systematic review and meta-analysis. Clin Nutr.

[104] Thedinga HK, Zehl R, Thiel A (2021). Weight stigma experiences and self-exclusion from sport and exercise settings among people with obesity. BMC Public Health.

[105] • Pearl RL, Puhl RM. Weight bias internalization and health: a systematic review. Obes Rev. 2018;19(8):1141–1163. **This systematic review identified 74 studies examining the association between weight bias internalization and mental and physical health outcomes. A strong negative association between weight bias internalisation and mental health was reported in the literature but there were fewer studies that examined physical health outcomes, and their findings were inconsistent.** 10.1111/obr.12701 PMC6103811 29788533

[106] Bayer R, Stuber J (2006). Tobacco control, stigma, and public health: rethinking the relations. Am J Public Health.

[107] Gee GC, Ro A, Gavin A, Takeuchi DT (2008). Disentangling the effects of racial and weight discrimination on body mass index and obesity among Asian Americans. Am J Public Health.

[108] Muennig P (2008). The body politic: the relationship between stigma and obesity-associated disease. BMC Public Health.

[109] Tomiyama AJ, Carr D, Granberg EM, Major B, Robinson E, Sutin AR, Brewis A (2018). How and why weight stigma drives the obesity ‘epidemic’ and harms health. BMC Med.

[110] Björntorp P, Rosmond R (2000). Obesity and cortisol. Nutrition.

[111] Girod JP, Brotman DJ (2004). Does altered glucocorticoid homeostasis increase cardiovascular risk?. Cardiovasc Res.

[112] Duru OK, Harawa NT, Kermah D, Norris KC (2012). Allostatic load burden and racial disparities in mortality. J Natl Med Assoc.

[113] Miller HN, LaFave S, Marineau L, Stephens J, Thorpe RJ (2021). The impact of discrimination on allostatic load in adults: an integrative review of literature. J Psychosom Res.

[114] O’Donoghue G, Cunningham C, King M, O’Keefe C, Rofaeil A, McMahon S (2021). A qualitative exploration of obesity bias and stigma in Irish healthcare; the patients’ voice. PLoS ONE.

[115] Phelan SM, Burgess DJ, Yeazel MW, Hellerstedt WL, Griffin JM, van Ryn M (2015). Impact of weight bias and stigma on quality of care and outcomes for patients with obesity. Obes Rev.

[116] Huizinga MM, Cooper LA, Bleich SN, Clark JM, Beach MC (2009). Physician respect for patients with obesity. J Gen Intern Med.

[117] Huizinga MM, Bleich SN, Beach MC, Clark JM, Cooper LA (2010). Disparity in physician perception of patients’ adherence to medications by obesity status. Obesity (Silver Spring).

[118] Foster GD, Wadden TA, Makris AP, Davidson D, Sanderson RS, Allison DB, Kessler A (2003). Primary care physicians’ attitudes about obesity and its treatment. Obes Res.

[119] Bertakis KD, Azari R (2005). The impact of obesity on primary care visits. Obes Res.

[120] Hebl MR, Xu J (2001). Weighing the care: physicians’ reactions to the size of a patient. Int J Obes Relat Metab Disord.

[121] Alberga AS, Edache IY, Forhan M, Russell-Mayhew S. Weight bias and health care utilization: a scoping review. Prim Health Care Res Dev. 20, E116. 10.1017/S1463423619000227 PMC6650789 32800008

[122] Alessi J, de Oliveira GB, Erthal IN, Teixeira JB, Scherer GDLG, Jaeger EH, Schneiders J, Telo GH, Schaan BD, Telo GH (2021). Diabetes and obesity bias: are we intensifying the pharmacological treatment in patients with and without obesity with equity. Diabetes Care.

[123] Puhl R, Peterson JL, Luedicke J (2013). Motivating or stigmatizing? Public perceptions of weight-related language used by health providers. Int J Obes.

[124] Amy NK, Aalborg A, Lyons P, Keranen L (2006). Barriers to routine gynecological cancer screening for White and African-American obese women. Int J Obes.

[125] Gudzune KA, Bennett WL, Cooper LA, Bleich SN (2014). Perceived judgment about weight can negatively influence weight loss: a cross-sectional study of overweight and obese patients. Prev Med.

[126] Puhl R, Brownell KD (2001). Bias, discrimination, and obesity. Obes Res.

[127] Roehling MV (1999). Weight-based discrimination in employment: psychological and legal aspects. Pers Psychol.

[128] Pingitore R, Dugoni BL, Tindale RS, Spring B (1994). Bias against overweight job applicants in a simulated employment interview. J Appl Psychol.

[129] Lee H, Ahn R, Kim TH, Han E (2019). Impact of obesity on employment and wages among young adults: observational study with panel data. Int J Environ Res Public Health.

[130] Sanburn J: Too big to cocktail? Judge upholds weight discrimination in the workplace. In*.* TIME: Time Magazine. 2013.

[131] Zee Rvd: Demoted or dismissed because of your weight? The reality of the size ceiling. In*.* The Guardian: The Guardian. 2017.

[132] Burris S (2002). Disease stigma in US public health law. Journal of Law, Medicine & Ethics.

[133] Roberto CA, Swinburn B, Hawkes C, Huang TTK, Costa SA, Ashe M, Zwicker L, Cawley JH, Brownell KD (2015). Patchy progress on obesity prevention: emerging examples, entrenched barriers, and new thinking. The Lancet.

[134] Keleher H, Murphy B (2004). Understanding health: a determinants approach.

[135] Mata J, Hertwig R (2018). Public beliefs about obesity relative to other major health risks: representative cross-sectional surveys in the USA, the UK, and Germany. Ann Behav Med.

[136] Kleinert S, Horton R (2015). Rethinking and reframing obesity. The Lancet.

[137] Daníelsdóttir S, O’Brien KS, Ciao A (2010). Anti-fat prejudice reduction: a review of published studies. Obes Facts.

[138] Sharma AM, Ramos Salas X (2018). Obesity prevention and management strategies in Canada: shifting paradigms and putting people first. Curr Obes Rep.

[139] Alberga AS, Pickering BJ, Alix Hayden K, Ball GDC, Edwards A, Jelinski S, Nutter S, Oddie S, Sharma AM, Russell-Mayhew S (2016). Weight bias reduction in health professionals: a systematic review. Clinical Obesity.

[140] Jensen MD, Ryan DH, Apovian CM, Ard JD, Comuzzie AG, Donato KA, Hu FB, Hubbard VS, Jakicic JM, Kushner RF (2014). 2013 AHA/ACC/TOS guideline for the management of overweight and obesity in adults: a report of the American College of Cardiology/American Heart Association Task Force on Practice Guidelines and The Obesity Society. Circulation.

[141] Ingram DD, Mussolino ME (2010). Weight loss from maximum body weight and mortality: the Third National Health and Nutrition Examination Survey Linked Mortality File. Int J Obes.

[142] Nimptsch K, Konigorski S, Pischon T (2019). Diagnosis of obesity and use of obesity biomarkers in science and clinical medicine. Metabolism.

[143] Koolhaas CM, Dhana K, Schoufour JD, Ikram MA, Kavousi M, Franco OH (2017). Impact of physical activity on the association of overweight and obesity with cardiovascular disease: The Rotterdam Study. Eur J Prev Cardiol.

[144] Knoops KTB (2004). de Groot LCPGM, Kromhout D, Perrin A-E, Moreiras-Varela O, Menotti A, van Staveren WA: Mediterranean diet, lifestyle factors, and 10-year mortality in elderly European men and women the HALE project. JAMA.

[145] Puhl R, Suh Y (2015). Health consequences of weight stigma: implications for obesity prevention and treatment. Curr Obes Rep.

[146] Łuczyński W, Głowińska-Olszewska B, Bossowski A (2016). Empowerment in the treatment of diabetes and obesity. J Diabetes Res.

[147] Puhl R, Peterson JL, Luedicke J (2013). Fighting obesity or obese persons? Public perceptions of obesity-related health messages. Int J Obes.

[148] Nestle M, Jacobson MF (2000). Halting the obesity epidemic: a public health policy approach. Public Health Rep.

[149] Ebbeling CB, Pawlak DB, Ludwig DS (2002). Childhood obesity: public-health crisis, common sense cure. The Lancet.

[150] Pérez-Escamilla R, Lutter CK, Rabadan-Diehl C, Rubinstein A, Calvillo A, Corvalán C, Batis C, Jacoby E, Vorkoper S, Kline L (2017). Prevention of childhood obesity and food policies in Latin America: from research to practice. Obes Rev.

[151] Caballero B: Humans against obesity: who will win? Advances in Nutrition. 2019, 10(suppl_1):S4-S9. 10.1093/advances/nmy055 PMC6363526 30721956

[152] O’Brien KS, Puhl RM, Latner JD, Mir AS, Hunter JA (2010). Reducing anti-fat prejudice in preservice health students: a randomized trial. Obesity (Silver Spring).

[153] MacLean L, Edwards N, Garrard M, Sims-Jones N, Clinton K, Ashley L (2009). Obesity, stigma and public health planning. Health Promot Int.

[154] Vallgårda S, Nielsen MEJ, Hansen AKK, Cathaoir KÓ, Hartlev M, Holm L, Christensen BJ, Jensen JD, Sørensen TIA, Sandøe P (2017). Should Europe follow the US and declare obesity a disease?: a discussion of the so-called utilitarian argument. Eur J Clin Nutr.

[155] Hofmann B (2016). Obesity as a socially defined disease: philosophical considerations and implications for policy and care. Health Care Anal.

[156] De Lorenzo A, Gratteri S, Gualtieri P, Cammarano A, Bertucci P, Di Renzo L (2019). Why primary obesity is a disease?. J Transl Med.

[157] Barber TM (2018). Is obesity a disease?. Expert Rev Endocrinol Metab.

[158] Hoyt CL, Burnette JL, Auster-Gussman L (2014). “Obesity is a disease”: examining the self-regulatory impact of this public-health message. Psychol Sci.

[159] Ramos Salas X, Alberga AS, Cameron E, Estey L, Forhan M, Kirk SFL, Russell-Mayhew S, Sharma AM (2017). Addressing weight bias and discrimination: moving beyond raising awareness to creating change. Obes Rev.

[160] Hilbert A, Hübner C, Schmutzer G, Danielsdottir S, Brähler E, Puhl R (2017). Public support for weight-related antidiscrimination laws and policies. Obes Facts.

[161] • Puhl RM, Lessard LM, Pearl RL, Grupski A, Foster GD. Policies to address weight discrimination and bullying: perspectives of adults engaged in weight management from six nations. Obesity (Silver Spring). 2021;29:1787–1798. **This study surveyed 13,996 adults taking part in an international weight-management programme, from Australia, Canada, France, Germany, the UK, and the USA. It found that these participants strongly supported laws and policies to address weight-based bullying and to address weight discrimination in workplace hiring.** 10.1002/oby.23275 PMC8571064 34612007

